# Stereotaktische Radiotherapie plus Nivolumab bei NSCLC

**DOI:** 10.1007/s00066-023-02143-0

**Published:** 2023-08-29

**Authors:** David Krug, Jürgen Dunst

**Affiliations:** Klinik für Strahlentherapie/Radioonkologie, UKSH, Campus Kiel, Kiel, Deutschland

## Hintergrund

Dass die stereotaktische Bestrahlung („stereotactic ablative body radiotherapy“ [SABR]) bei nichtkleinzelligem Bronchialkarzinom (NSCLC) im Stadium I eine exzellente Therapieoption darstellt, ist unstrittig; die gepoolten Daten der beiden zwar sehr kleinen, aber randomisierten Studien (STARS und ROSEL, zusammen *N* = 58) zeigen sogar einen Überlebensvorteil der SABR gegenüber einem operativen Vorgehen [1]. Bisher ist es uns Radioonkologen aber nicht gelungen, diese Evidenz in eine eindeutige allgemeine Therapieempfehlung umzusetzen. Die anderen Disziplinen verweisen nämlich auf etwa ein Dutzend Metaanalysen nichtrandomisierter Studien und Kohortenanalysen großer Datenbanken (z. B. SEER) mit teilweise über 10.000 Patienten, die einen Überlebensvorteil für operierte Patienten ausweisen. Allerdings zeigen diese nichtrandomisierten Studien keinen Vorteil in der lokalen Kontrolle und im krankheitsspezifischen Überleben, sodass ein Bias durch Patientenselektion vermutet werden muss (z. B. [3, 4]). In der Konsequenz gilt die operative Therapie immer noch als Option der 1. Wahl bei operablen Patienten sowohl in der deutschen S3-Leitlinie als auch in internationalen Empfehlungen [5, 6]. In der aktuellen hier referierten Studie des MD Anderson Cancer Center (MDACC) wurde gezeigt, dass die Ergebnisse der SABR durch eine zusätzliche Immuntherapie weiter verbessert werden können [2]. Diese Daten könnten die Diskussion um die optimale Therapie weiter beleben.

## Patienten und Methodik

In dieser randomisierten Studie, die an drei Kliniken in Texas unter Leitung des MDACC durchgeführt wurde, konnten Patienten mit histologisch gesichertem NSCLC bis maximal 7 cm Durchmesser ohne LK-Befall (N0 M0) aufgenommen werden, auch isolierte Lokalrezidive. Für das Staging war eine PET-CT obligat, und eine Abklärung der mediastinalen LK durch transbronchiale Biopsie (LK-Stationen 4 und 7 beidseits) oder Mediastinoskopie wurde dringend empfohlen und war obligat bei Lymphknoten mit einem Querdurchmesser von über 1 cm. Wichtigstes Ausschlusskriterium neben Tumorgröße war eine zentrale Tumorlage (weniger als 5 mm Abstand zu den Risikostrukturen Trachea, Herz, Ösophagus, große Gefäße, Plexus; nebenbei: in der aktuellen deutschen Leitlinie wird ein Abstand von 2 cm vom Tracheobronchialbaum empfohlen). Operabilität war kein Ausschlusskriterium. Nach Randomisierung erhielten die Patienten entweder nur eine stereotaktische RT (SABR-Gruppe) oder eine stereotaktische RT plus Nivolumab (I-SABR-Gruppe). Die Radiotherapie erfolgte nach 4‑D-CT-Planung mit vier Fraktionen mit 50 Gy im PTV bzw. 60 Gy im GTV oder mit 10 Fraktionen mit 70 bzw. 80 Gy. Die Therapie mit Nivolumab (480 mg alle 4 Wochen, insgesamt 4 Zyklen) startete am Tag der ersten RT oder innerhalb von 36 h.

## Ergebnisse

Von Juni 2017 bis März 2022 wurden 156 Patienten randomisiert (78 pro Gruppe, ITT-Population), und 141 wurden als Per-protocol-Population (75 SABR, 66 I-SABR) ausgewertet. 28 Patienten hatten ein Lokalrezidiv; 32 Patienten galten als operabel, hatten eine Op. aber abgelehnt. Nach einem medianen Follow-up von 38 Monaten betrug das ereignisfreie 4‑Jahres-Überleben in der I‑SABR-Gruppe 77 % gegenüber 53 % in der SABR-Gruppe (HR 0,38, *p* = 0,0056; in der ITT-Population war der Effekt ähnlich mit HR = 0,42, *p* = 0,008). Der Effekt der zusätzlichen Immuntherapie (gemessen als Hazard Ratio) war in der Subgruppenanalyse in allen Untergruppen relativ ähnlich, obwohl die Ergebnisse bei Patienten mit Tumoren größer als 2 cm (HR 0,40) oder Rezidiven (HR 0,52) formal keine Signifikanz erreichten. Bei Patienten mit positivem PD-L1-Status (PD-L1-Expression > 1 %) trat in beiden Gruppen kein Lokalrezidiv auf. Die zusätzliche Immuntherapie wirkte auch bei Patienten mit negativem PD-L1-Status sehr gut (HR 0,26; allerdings war der PD-L1-Status nur bei 65 % der Patienten bestimmt worden). Bezüglich der Rezidivraten wurden in der SABR-Gruppe 13 % Lokalrezidive, 11 % regionale Rezidive und 16 % Fernmetastasen beobachtet, in der I‑SABR-Gruppe waren das 0 %, 6 % und 3 %. Neue Lungentumoren wurden bei 8 % bzw. 3 % beobachtet, Todesfälle in 12 % bzw. 6 %. Bezüglich der Toxizität gab es keine relevanten Nebenwirkungen (Grad 3 oder höher) durch die SABR, aber 15 % Grad-3-Toxizitäten in der I‑SABR-Gruppe, von denen jedoch alle reversibel waren.

## Bewertung der Autoren

Eine kurzeitige (dreimonatige) simultane Immuntherapie mit Nivolumab verbessert die Ergebnisse der SABR hochsignifikant, insbesondere die lokale Tumorkontrolle.

## Kommentar

Trotz der präliminären Daten und der kleinen Patientenzahl ist das eine exzellente Studie. Folgende Punkte erscheinen uns beachtenswert:

1. Die im Verhältnis zu anderen Studien schlechten Ergebnisse im ereignisfreien Überleben sind durch die sehr großzügigen Einschlusskriterien (weit überwiegend inoperable Patienten, z. T. große Tumoren oder Rezidive) erklärbar.
2. Bei Patienten mit positivem PD-L1-Status war die lokale Kontrolle 100 %, mit und ohne Immuntherapie. Das kann man nicht mehr überbieten. Auch sonst ist die lokale Effektivität der SABR hoch, sodass die Kombination mit einer Immuntherapie für manche Patienten mit günstiger Ausgangslage (Ersterkrankung, kleiner Tumor) eine Übertherapie darstellen kann.
3. Die Immuntherapie hatte einen eindrucksvollen Effekt, obwohl sie relativ kurz war (nur 4 Infusionen, also kein Vergleich zu einer Erhaltungstherapie mit beispielsweise Durvalumab über ein Jahr im Stadium III).
4. Der Effekt der Immuntherapie betraf alle Rezidivlokalisationen (lokal, regional, distant, Zweitkarzinome), war aber besonders eindrucksvoll bezüglich der lokalen Kontrolle. Das stärkt die Hypothese von synergistischen Effekten. Die zeitliche Abfolge der Therapieverfahren könnte eine ganz entscheidende Rolle spielen. Der Stellenwert der adjuvanten Immuntherapie nach Resektion ist bisher nicht eindeutig geklärt. Atezolizumab wurde für die adjuvante Therapie nach platinbasierter Chemotherapie bei hohem Rezidivrisiko und PD-L1-Expression ≥ 50 % auf den Tumorzellen zugelassen. Im Stadium IIA/B zeigte sich in der IMpower010-Studie allerdings kein signifikanter Vorteil in Bezug auf das krankheitsfreie Überleben [7]. Pembrolizumab verbesserte in der PEARLS/KEYNOTE-091-Studie das krankheitsfreie Überleben, dies galt jedoch nicht für das Stadium IB [8].
5. Die Immuntherapie ist beim NSCLC sehr effektiv. Die bisher größte Effektivität (gemessen an der Hazard Ratio) wurde aber in dieser Studie beobachtet.
6. Die Toxizität der zusätzlichen Immuntherapie erscheint zunächst moderat und ist sicherlich auch durch die kurze Behandlungsdauer begünstigt. Nichtsdestotrotz muss berücksichtigt werden, dass insbesondere funktionell inoperable Patienten eine hohe Komorbidität aufweisen und somit ein besonders vulnerables Patientenkollektiv darstellen. In der vergleichbaren, kürzlich im *Red Journal* publizierten iSABR-Phase-1-Studie, die eine Kombination von SABR und Durvalumab untersuchte, entwickelten 5 von 18 Patienten pulmonale Toxizitäten Grad ≥ 3, hierunter 4 Pneumonitiden, von denen eine tödlich verlief [9].

Fazit: Für diese Indikation gibt es bisher keine Zulassung der Immuntherapie, und die praktische Umsetzung ist deshalb limitiert. Der Einfluss auf das Gesamtüberleben sowie die Daten aus weiteren laufenden randomisiert-kontrollierten Studien (u. a. KEYNOTE-867, PACIFIC‑4, SWOG/NRG S1914) bleiben abzuwarten. Trotzdem stärkt diese Studie die Position der SABR, und die exzellente lokale Kontrolle bei PD-L1-positiven Tumoren mit alleiniger SABR (kein Lokalrezidiv!) kann man schon heute als Argument nutzen. Zukünftig sollte vermehrt auch bei funktionell nichtoperablen Patienten eine histologische Sicherung erreicht werden, auch wenn diese nach aktuellem Stand der S3-Leitlinie bei typischer CT-Morphologie und FDG-Utilisation sowie mindestens Größenpersistenz über ≥ 4 Wochen nicht zwingend gefordert wird.

*David Krug und Jürgen Dunst, Kiel*
